# Extending computational models of astrocyte-neuron interactions with biochemical mechanisms on the postsynaptic terminal

**DOI:** 10.1186/1471-2202-16-S1-P148

**Published:** 2015-12-18

**Authors:** Aušra Saudargienė, Tiina Manninen, Riikka Havela, Marja-Leena Linne

**Affiliations:** 1 Department of Informatics, Vytautas Magnus University, Kaunas, Lithuania; 2 Department of Signal Processing, Tampere University of Technology, Tampere, Finland

## 

There is increasing evidence that astrocytes not only interact with each other but also with adjacent neurons in the neural circuitry in a variety of brain areas. Many biophysical and biochemical mechanisms have been proposed to explain these interactions *in vitro*. Based on the experimental literature, the mechanisms involved seem to depend not only on the developmental stage of an animal but also on the brain area, neural circuitry, as well as on the experimental technique used to characterize the phenomena. Using biophysically and biochemically detailed compartmental models, we are interested in understanding how these interactions between neurons and astrocytes may regulate information processing and, notably, different forms of synaptic plasticity [1]. In this study, we designed a new model of the so-called tripartite synapse. The tripartite synapse is a concept of synaptic physiology in which there are three parts of a synapse: the presynaptic and postsynaptic terminals, and an astrocyte in between them [2]. Many previous studies *in vitro *have shown that gliotransmitters like glutamate are released from astrocytes into the synaptic cleft following activation of presynaptic or postsynaptic terminals, or activation of the astrocyte itself [3]. However, none of the previous modeling studies incorporate all features necessary to understand the complex interactions in full detail. We extend beyond these previously published fundamental, yet relatively simplistic representations (see, e.g., [4]). We describe the postsynaptic terminal using a two-compartmental approach, in addition to the presynaptic terminal and the astrocyte. We specifically address the postsynaptic mechanisms and their role in activating astrocytic calcium signaling and subsequent gliotransmission. By introducing realistic model components on the postsynaptic terminal, such as voltage-dependent receptor channels and the G-protein activated signaling cascades, we observe stimulus-dependent changes in astrocyte calcium oscillations leading to activation of not only astrocytes, but also of adjacent neurons. The long-term goal of our work is to develop detailed models of astrocyte-neuron interactions for different brain areas which allow testing experimentally evoked hypothesis, as well as some controversies in the field (see, e.g., [5]). These models will open up new avenues to assess the effects of numerous mechanisms of astrocytes on the dynamics of local neuronal networks. Only through systematic integration of *in vitro *and *in silico *work will we be able to understand how astrocytes may contribute to brain information processing and plasticity.
